# What Propels The Pharma Industry?

**DOI:** 10.4103/0973-1229.32156

**Published:** 2007

In this final section, we shall see what are the ground realities that both industry and academia-biomedicine have to acknowledge; what are the major propellants of the pharma industry; what is the difference between marketing a pharma and any other product; how tactics of the short term industry player, although attractive, are unsuited for the long term player; where is secrecy justified and where not; why does pharma adopt questionable means at all; what are the four different types of drugs according to usefulness and side-effects; and what is a reasonable blueprint for industry to follow in an attempt to re-establish credibility without forsaking profits.

## Accept Some Ground Realities

A rethink on the academia-industry connect is only possible if some ground realities are clearly accepted:

### Understanding what propels pharma and its constraints

We all accept that industry profits have to be made and investment in research and development in the pharma industry is massive. R and D costs are staggering and several studies put the cost of researching and developing a new chemical entity (NCE) at Euro 895 million (EFPIA, 2007). Research driven pharma companies invest about 15–20% of their sales in R and D, which is higher than any other industrial sector, including high tech industries such as electronics, aerospace or automobiles (ibid). We all also realise that, on an average, one new drug come from the laboratory to the pharmacy shelf after 12 to 13 years; and it is after 5000 to 10000 promising ones are shelved due to extensive testing in the R and D phase that a drug is approved as a quality, safe and efficient marketable product (ibid). Moreover, the price of a new medicine carries a contribution towards the cost of discovering the next (ibid). This too is an add-on to the MRP.

### Difference in pharma and other marketing

However, we must accept there is a fundamental difference between marketing strategies for other entities and those for drugs. If the former do not work, the market will simply reject them after an initial upsurge due to hype. Another new product can be hyped and launched and profits made. And the game played on endlessly. Such a process cannot, however, be allowed or justified for a drug. Simply because if a drug does not work or has serious side-effects, it is not just a matter of market rejection, but can result in serious complications: exacerbation of disease, morbidity, even mortality. We have discussed the case of aprotinin (p38–40), alteplase (p79–80) and rhAPC (p45–47) in this connection. Hence, so-called valid marketing techniques in other fields cannot be justified here. Market savvy executives and smart market players, may avoid such realization at their own peril. And it is the task of enlightened researchers and journals and their editors, that such games playing get ruthlessly exposed.

### Long term and short term industry players

What industry captains and CEOs have to realize is that you can hype products, induce doctors by fringe benefits, use pliant researchers to fill journal pages, have impressive spokespersons to impress audiences, manipulate evidences and performance criteria, enter CPG Bundles or whatever: but how can you get the ultimate beneficiary, the patient, well, unless your drug works? And how can you conceal side-effects and adverse drug reactions when they do occur? No hype or tall talk or journal pages can ensure that. Either the drug works or it doesn’t. Either it has serious side-effects or it doesn’t. The ultimate barometer is patient welfare and no drug that compromises it can stand the test of time. So, how does it make even commercial sense in the long term to market substandard products or compromise research integrity? Short term, possibly, yes. But those who are long-term players must realize they play this game with such market savvy techniques at their won peril. It is possible short-term (and new upstarts) may adopt such techniques to upstage established players and appear to prosper while so doing. But even they cannot prosper for long unless they consider patient welfare and scientific evidence. They will only remain fringe players otherwise; and never establish credibility, so what some profits get made initially; and they will often face litigation and other hassles.

### Tactics of the short-term players unsuitable for the long-term player

The greatest mistake long-term players in industry may make is try to adopt the shady techniques of the upstart new entrant. They may appear to win market share initially due to this and may even temporarily decimate opposition. What is more likely to happen is they may get enamoured of the fancy techniques of such upstarts and stake their well earned reputation and credibility for the quick buck. The upstart will make his bucks and disappear. He can even afford to be a fly-by-night operator; he may even be one. The long-term player has nowhere to go. The upstart has no notions of earning sustained credibility. The long-term player survives only because of it. So, it is suicidal for the long-term player to adopt smart but unethical market strategies of the upstart, strategies having scant regard for established scientific norms in research and commitment to patient welfare. And it is suicidal for them to encourage ‘intrusion of marketing strategies masquerading as evidence based medicine’ (Eichacker *et al.* 2006), for they will be exposed eventually and their credibility cannot but take a severe beating as a result. This realization must sink clearly into our minds.

### Secrecy, justified and unjustified

Secrecy of marketing/sales tactics, of the process of manufacture, of other strategies and plans of business expansion, of strategies to tackle competition etc are fine business tactics. Possibly. But it is critical that secrecy as a tactic does not extend to research findings, especially those contrary to one’s product. Such secrecy is all the more attractive to follow for it guarantees the drug recovers costs (and, hopefully, also makes handsome profits) before it falls by the way side; which in any case it will, since its poor market potential is known anyway. (And drugs nowadays fall out of favour quickly even if they are effective: so why divulge adverse/negative reports at all?) Such secrecy may also unwittingly become a marketing ploy since such is the undercurrent all through the organization. But it’s not likely to succeed, precisely because, rather than work for the company’s good, it works to expose the company to adverse publicity and litigation, both of which are ominously on the rise in the last few years, besides reducing credibility and public support.

### Moral

So, moral of the story: you have no option but to make a quality product, do comprehensive adverse reaction profile and market only if it passes both tests. Any attempt to hoodwink people is not likely to succeed any longer, what with heightened awareness of wrong doing all around.

## Why Does Pharma Adopt Questionable Means At All?

The question still remains. Why does pharma adopt questionable means at all? Agreed one spends a fortune making a drug. One can find means and men to trumpet its qualities in journals and sponsored programmes and jaunts. One can create appropriate media hype and pamper end users, the prescribers, to go gaga over the new drug. But ultimately the drug has to work and not cause serious side-effects. It’s not possible to gloss over these by any marketing techniques. Or is it?

The answer to this is not easy, but let’s try.

### Pharma business cannot depend only on genuine discoveries

Look at it from the industry angle. What with all the constraints, a drug comes to the pharmacy after huge investments. There are crippling overheads and infrastructure costs to be recovered. And there are massive profit margins to be maintained. If these were to be dependent only on genuine drug discoveries, that would be taking too great a risk. Rather, it makes more business sense to promote block-busters and masquerade them as genuine discoveries. The difference between a block-buster and a genuine discovery is that the first is a creation of the marketing department while the latter is of the research department. A block-buster is always needed. If it’s a genuine discovery, so much the better; if not, they hype it to appear as one. In any case, whether genuine or a ‘me-too’, any new drug has a short dream run on a market ever thirsting for novelty and hype. Does it not then make sense to market a number of potential winners and back them to the hilt? Some will be real champions, some will go bust and some will survive due to the hype. All, however, will rake in the dollars. Moreover, the placebo effect of a new hyped drug can be strong. Before its time to evaluate whether the drug really works or not, there is already a new entrant launched and the earlier one just fades off. And the game continues endlessly.

### Newer drugs with no effect are also with no or little, side-effects - that helps

Moreover, with regard to the drugs themselves, it’s of course best to have those which work and have least side-effects. But it’s equally wonderful to have drugs with least side-effects which hardly work. Now these may be little better than placeboes. (In fact most drugs, before they are marketed, are compared with placeboes alone, while, actually speaking, they should be compared with existing ‘gold standards’ - something which needs speedy correction.) When they get written by prescribing doctors, they often replace existing medications. Since they have no (or less) side-effects and since the earlier one did have them, patients may feel great relief. Not because of the new drug, but simply because the earlier one was withdrawn. When it’s time to judge the real effect, it’s time to be excited about a new drug launch in the same category. So the game continues.

### Image building helps balance profit with shored up credibility

Industry players have to strike the right balance between profit making and credibility. In profit making, the marketing champions play their role. In credibility ratings, researchers and paid spokes-persons play their role. All is hunky dory till marketing is based on credibility. When there is nothing available to make for credibility, something is projected as one and marketing carried out, in the calculated hope that profits can accrue, since profit making must continue endlessly. That is what makes pharma adopt even questionable means to make profits.

To realize why this happens is not to defend it. The critical question is for pharma to ask itself: what can it do so it establishes well-founded credibility even while making profits? To understand this, we must know what is the type and potential of each drug.

## Four Types Of Drugs

Essentially, there are four types of drugs. First, drugs that work and have minimal side-effects; second, drugs which work but have serious side-effects; third, drugs that do not work but have minimal side-effects; and fourth, drugs which work minimally but have serious side-effects.

### Effective and not dangerous: The real champions

Drugs which work and have minimal side-effects are great. They are the ideal of every researcher and dream of every pharma company. But they are few and far between, therefore cannot sustain huge infrastructure and overhead costs and cannot generate the desperately needed huge profits. They are the champions that go on to become the gold standards in their respective categories. They raise credibility effectively.

### Effective, but dangerous: The potential champions who could not make it

The second type of drugs are those that work but have serious side-effects. Well, they too are needed since the first type are few and far between. And they always help create a demand for newer molecules with fewer side-effects, a genuine need that can, however, also be exploited as a smart marketing technique. They are the ones which can create problems for the manufacturer in the form of adverse publicity and litigation. But they also help create a felt need and niche for drugs which may not work but have minimal side-effects: the third type which we can now look into.

### Not effective and not dangerous: The projected trail blazers who fade away

These are the clone types, the placeboes, the ‘me-toos’, drugs which are, well, not great, but equally necessary to fill up coffers. Smart market players hype them with clever inputs from market savvy scientists. They have a dream run like the pace setter in a marathon race. They blaze the track and just fade away after a few rounds. But their job is done. They bleed themselves for their profit masters and get thrown by the wayside. But they achieve their potential, since they fill up important gaps till the real champions get discovered and can get into the act. Or, a new pace setter can be found to carry on the race in the absence of a real champion.

### Not effective and dangerous: The problematic laggards

The last type are those which work minimally but have serious side-effects. These are the real bleeders for industry. They bleed profit margins dry. Smart operators quickly forsake these. They get into the stable either by accident or by errors of omission. Some smart operator knows there are side-effects but conceals them to launch a new drug to add to the tally, hoping that market hype and pliant end-users will carry the day, at least for a while. These are the real problem, for they run huge litigation risks and involve major credibility problems.

## Hassles and The Cat And Mouse Game

It is the second and fourth types that create major hassles for industry. Often, industry may try to project the fourth type as the second to escape censure. The major cat and mouse game being played by conscientious researchers is in exposing the third and fourth for what they are and not allowing industry to palm them off as the first and second type respectively. The other major game is in preventing the second type from being projected as the first. The third are essentially harmless, so they attract censure all right, but escape anything more than a light tap on the knuckle and some merriment at the antics to market them, except when they are projected as the first type.

## Some Interesting Realisations and Solutions

What is necessary is for industry captains and long-term players to realise:

1. Their major propelling force can only be producing the first type. All hype to promote and market them is justified. Industry players can justifiably go overboard in trying to produce and market such discoveries. Industry watchers should beware they not go overboard in trying to shoot down such discoveries.
2. They accept the second type only till they can lay their hands on the first and, in any case, never project or accept, them as the first.
3. The third type can be occasionally played around with to shore up profits, but never by projecting them as the first type, never at the cost of forgetting to produce the first, and never promoting them in conditions which can be morbid and potentially fatal.
4. The fourth type are the laggards and a real threat to credibility and therefore do not deserve any market hype or promotion. What they deserve is a quiet burial.

In finding out why most pharma indulges in questionable tactics, we are lead to some interesting solutions to prevent these tactics with the least amount of hassles for all concerned. In following them not only do they salvage lost credibility, they also ensure long-term presence of conscientious industry players and continued inflow of profits. A better formula may be possible of course, but till it is found, this may work well for most if they apply their minds to it.

## Concluding Remarks

The process of self-correction set into motion due to greater clout of conscientious researchers, unrelenting expose by medical journalists and supportive editors, patients right activism and law suits against industry will, hopefully, help tilt the balance towards value-based advance, even if belated, and done grudgingly. Major industry players may soften the offensive of such self-correction only by playing the game according to the rules. The earlier the major players understand this, the better it is for all concerned.
